# Experience of lanadelumab administration in hereditary angioedema: A case series of 4 patients in Portugal

**DOI:** 10.5415/apallergy.0000000000000102

**Published:** 2023-06-06

**Authors:** Ana Graça Bernardino, Manuel Branco Ferreira, Célia Costa, Joana Caiado, Elisa Pedro, Amélia Spínola Santos

**Affiliations:** 1 Serviço de Imunoalergologia, Hospital Santa Maria, Centro Hospitalar Universitário de Lisboa Norte, Lisboa, Portugal; 2 Clínica Universitária de Imunoalergologia, Centro Académico de Medicina de Lisboa. Faculdade de Medicina, Universidade de Lisboa, Lisboa, Portugal

## To the Editor,

Hereditary angioedema (HAE) is a rare autosomal dominant disease, which is mostly caused by reduced levels or dysfunctional activity of the serine protease inhibitor C1-inhibitor (C1-INH) [1, 2]. HAE patients present transient swelling episodes affecting subcutaneous and/or submucosal tissues, mainly involving skin, abdomen and upper respiratory tract, which in some cases can be fatal [1–3]. Since HAE symptoms recur with unpredictable frequency and severity throughout patients’ lives, effective prevention of attacks is essential in patients’ care [4]. Long-term prophylaxis (LTP) has the goal of achieving complete disease control in order to normalize patients’ lives [1, 2]. According to national recommendations and the latest WAO/EAACI guidelines, LTP should be individualized and considered in all HAE patients, taking into account disease activity, patient’s quality of life (QoL), availability of healthcare resources and failure to achieve adequate control by optimal on-demand therapy [1–3].

Lanadelumab, a human monoclonal antibody inhibitor of plasma kallikrein, is currently considered a first-line LTP for HAE in patients ≥12 years of age (excluding pregnancy [1, 5]), supported by pivotal and open-label extension studies, that showed marked reduction of acute attacks and improvement in patients’ QoL [6].

Since 2020, the use of lanadelumab for LTP was authorized by the Portuguese Health Authority under an early access program (EAP) for HAE patients showing contraindication or insufficient efficacy of LTP with intravenous C1 inhibitor concentrate. In Portugal, EAP aims to use medicines, without marketing authorization or that still have no prior reimbursement assessment decision of the national healthcare system, that could be used in the treatment of certain pathologies where there is no demonstrated therapeutic alternative for a particular patient. The procedure requires a special authorization request for each eligible patient, by the hospital institution where the medicine will be used [7]. Since February 2022, lanadelumab is fully reimbursed by the Portuguese Health System for routine prevention of recurrent HAE attacks in HAE patients > 12 years of age, with contraindication or the absence of control with androgens and/or antifibrinolytics [8]

Our aim was to describe our first HAE patients receiving lanadelumab under this EAP. This 2-year retrospective study of 4 HAE patients, enrolled in lanadelumab EAP in our department between June 2020 and September 2021, was approved by the Ethical Committee of our hospital. Demographic and clinical data were collected from the clinical files, including disease control and QoL scores, HAE acute attacks and disease severity classification, before lanadelumab (while patients were receiving their usual LTP) and in June 2022, under lanadelumab.

The HAE severity classification proposed by Agostoni et al [9]. was slightly adapted to also include icatibant as an option in emergency treatment. We used the Portuguese versions of angioedema control test (AECT) and angioedema quality of life instrument (AE-QoL) in the last 4 weeks [10, 11]. Disease control was considered if an AECT score >10 was registered. A minimal clinically important difference (MCID) on the AE-QoL score was considered if an improvement >6 points was observed [12, 13].

Demographic and clinical characteristics of the patients are described in Table 1. Four patients (3 females, 1 male) were included: 2 type 1 HAE and 2 with type 2. Mean age was 51 years; mean age of first HAE symptoms was 11 years and the mean age at diagnosis was 24 years.

**Table 1.** Demographic and clinical characteristics of hereditary angioedema patients before and after lanadelumab

			Patient 1					Patient 2					Patient 3					Patient 4				
Gender/age (y)			F/39					M/41					F/40					F/57				
HAE type			2					1					2					1				
Onset of symptoms (age)			Preadolescence (12 y)					Childhood (1 y)					Adolescence (18 y, with OC)					Adolescence (13 y)				
Age of diagnosis (y)			13					18					24					42				
Location of acute attacks			facial; laryngeal; Abd; extremities Asphyxia and OTI at 16 y					facial; laryngeal; Abd; genital; extremities. Ascites/Hospital admission					laryngeal; Abd; extremities Ascites/2 Abd surgeries (18, 23 y)					Cervical; trunk; Abd; genital; extremities. Appendectomy (15 y)				
Before lanadelumab	Previous LTP and side-effects		16–26 y: danazol 100–200 mg/d, stopped due to hepatic nodule 26–27 y: _iv_C1INH 4×/wk with partial efficacy (pregnancy) Since 27 y: under danazol 200 mg/d with amenorrhea 36 y: MRI: hepatic adenoma					18–39 y: danazol 200–400 mg/d 39 y: danazol 400 mg/d with increased liver enzyme levels Since 40 y: _iv_C1INH 2×/week with partial efficacy and poor venous accesses					24–37 y: danazol 200–400 mg/d with hirsutism and amenorrhea 37–39 y: _iv_C1INH 3–4×/wk with partial efficacy (pregnancy) Since 39 y: danazol 400–800 mg/d					42–55 y: danazol 400 mg/d with increase in liver enzyme levels 56 y: _iv_C1INH 2×/wk with low efficacy Since 57 y: under danazol 200 mg/d				
	Acute treatments		_sc_Icat or _iv_C1INH sos ≥ 2×/y					_sc_Icat or _iv_C1INH sos ≥ 1×/y					_sc_Icat sos ≥ 3×/y					_sc_Icat or _iv_C1INH sos ≥ 2×/y				
	AECT score		7					1					1					4				
	AE-QoL score: domains		24: 4; 4; 14; 2					51: 16; 8; 22; 5					53: 16; 13; 20; 4					37: 4; 11; 20; 2				
	Acute attacks in previous year		10					12					42					30				
	Severity classification (score)		Severe (47)					Severe (43)					Severe (85)					Severe (67)				
Lanadelumab started in (300 mg every 2 wks)			June 2020					December 2020					January 2021					September 2021				
After started lanadelumab	Stepdown LTP		Danazol stopped at 1st mo Lanadelumab monthly at the 12th mo					_iv_C1INH stopped at 1st mo Lanadelumab monthly at the 6th mo					Danazol stopped at 11th mo Lanadelumab every 2 wk					Danazol stopped at 8th mo Lanadelumab every 2 wk				
	AECT score		12					16					12					15				
	AE-QoL score: domains		4: 0; 0; 4; 0					6: 0; 0; 6; 0					9: 0; 4; 5; 0					4: 0; 0; 4; 0				
	Evolution androgen side-effects		Recovered menstrual cycles					Reduction in levels of hepatic enzymes					Recovered menstrual cycles					Reduction in levels of hepatic enzymes				
	Acute attacks evolution	Time	N	M	Invol	Trig	Treat	N	M	Invol	Trig	Treat	N	M	Invol	Trig	Treat	N	M	Invol	Trig	Treat
		0–6 mo	1	5	Abd	S	_sc_Icat	0	-	-	-	-	1	6	Abd	Inf	_sc_Icat	1	6	Face	DP	0
		6–12 mo	0	-	-	-	-	0	-	-	-	-	2	7/12	Abd	Inf/S	_sc_Icat/0	0	-	-	-	-
		12–18 mo	0	-	-	-	-	0	-	-	-	-	1	16	Abd	Inf	0					
		18–24 mo	1	19	Abd	S	_sc_Icat	0	-	-	-	-										
	Severity classification (score)		1 (31)					2 (25)					1 (37.5)					2(25.5)				

Abd, abdominal; AECT, angioedema control test; AE-QoL, angioedema quality of life; DP, dental procedure; Inf, infection; Invol, involvement; iv C1INH, intravenous concentrated C1 inhibitor; HAE, hereditary angioedema; LTP, long-term prophylactic treatment; M, month; MRI, magnetic resonance imaging; N, number; OC, oral contraception; OTI, orotracheal intubation; sc Icat, subcutaneous icatibant; S, stress; Treat, treatment; Trig, trigger.

Before lanadelumab, all 4 patients suffered from severe recurrent HAE acute attacks that affected mostly the abdomen (100%), extremities (100%), larynx (75%), face (50%), genitals (50%), and trunk and cervical area (25%). Patient 1 had an episode of asphyxia, needing orotracheal intubation. Patients 2 and 3 had recurrent severe abdominal acute attacks with ascites requiring hospital admissions. Patient 4 was submitted to appendectomy due to a misdiagnosed HAE acute severe abdominal attack. Mean number of acute attacks during the year before lanadelumab was 23.5, several of these attacks receiving on-demand treatment with _sc_Icatibant or _iv_C1-INH. All patients used androgens with associated adverse events (2 patients) or poor efficacy (2 patients). _iv_C1-INH was also used as LTP in all patients with only partial efficacy. In addition, patient 2 had poor venous accesses, rendering him noncompliant to repeated iv administrations.

Before lanadelumab all patients had poor disease control with AECT scores <8 (mean 3.25), and a high impact in QoL, at least for 3 patients (mean AE-QoL score of 41.25 points). At the end of June 2022, the duration of lanadelumab therapy ranged between 10 and 25 months with no severe adverse events reported. No patient stopped lanadelumab.

Regarding the intervals between lanadelumab administrations, patients 1 and 2 switched to a monthly administration at the 12th and 6th months, respectively. Patients 3 and 4 still maintained a 2-week interval administration, with gradual reduction of danazol, which was stopped, respectively, on the 11^th^ and 8^th^ months after the start of landelumab. Maintenance of danazol therapy for some additional months was due to the high degree of patients’ confidence on androgen therapy and to doctors’ and patients’ fear of attacks, which was reinforced by the presence of some attacks/symptoms in these patients. Nevertheless, after some months, patients could be convinced to successfully stop androgens.

All patients reported substantial reductions in the number of acute attacks. Patient 1 had two HAE attacks in 2 years; patient 2 had 0 attacks in 19 months, although having a mild episode of *erythema marginatum* in the 18th months of lanadelumab, which resolved spontaneously without any treatment; patient 3 had 4 attacks in 18 months and patient 4 had 1 attack in a 10-month period. No patient needed to be treated in an emergency department. Mean reduction of the number of HAE attacks was 94.75%. Moreover, in patients 1 and 2, the reduction of lanadelumab to one administration every 4 weeks was not associated with any clinical deterioration.

The score in the Agostoni et al severity scale showed an improvement in all patients. AECT increased in the 4 patients, all reaching scores indicating controlled disease (>10). Mean AECT score in June 2022 was 13.75. Additionally, we observed also a very significant improvement in AE-QoL score. In June 2022, mean AE-QOL score was 5.75 points. These were much lower values compared to pre-lanadelumab AE-QoL assessments, with an average improvement of 35.5 points. All patients had a reduction >6 points, meaning that with lanadelumab treatment all patients reached a minimal clinically important difference (MCID) in QoL. Within the 4 individual domains, it was possible to observe a complete resolution of the impact of the disease regarding “functioning” and “nutrition” domains in all patients and regarding “fatigue/mood” in 3 patients. Some aspects related to the domain “fear/ shame” were still being reported by all patients, however with improvement when compared with pre-lanadelumab values.

The results of this first series of Portuguese HAE patients treated with lanadelumab emphasize that LTP with lanadelumab is effective and well tolerated, resulting in a considerable reduction of HAE acute attacks, ranging between 90% and 100% reduction in acute attacks/years. Although with a very small number of patients, these results are in line with other phase IV studies, one with 212 patients and another with 93 patients, where reductions in attack rates of 87% and 83% were observed, respectively [6, 14]. Similarly, a Canadian series of 12 HAE patients, refractory to LTP with C1-INH concentrate, showed that lanadelumab could attain better disease control and minimize the burden of treatment, with a 72% reduction in attack rate [15]. We also observed a substantial improvement in AECT scores (mean value improvement from 3.25 to 13.75) and in AE-QoL scores (mean value reduction from 41.25 to 5.75). Our results are in line with a real-life German study with 34 HAE patients [16], where an improvement of AECT scores from 7.5 to 14.9 was observed, as well as a reduction of AE-QoL scores of 32 points (45.8–13.8 points).

Given the favorable results observed, we expect that LTP with lanadelumab can be more frequently used, allowing to discontinue androgen LTP in more HAE patients.

## Conflicts of interest

The authors have no financial conflicts of interest.
